# From rationality to identity: the impact of using community health services for the aged on the well-being of older adults in China

**DOI:** 10.3389/fpubh.2024.1447217

**Published:** 2024-09-24

**Authors:** Liu Yang, Lijian Wang

**Affiliations:** School of Public Policy and Administration, Xi’an Jiaotong University, Xi’an, China

**Keywords:** community health services for the aged, objective well-being, subjective well-being, older people, instrumental variable

## Abstract

**Objectives:**

To better understand the outcome benefits of community health services for the aged (CHSA) and provide bottom-up identity for development, this study examined the impact of using CHSA on well-being of older adults from both objective and subjective dimensions.

**Methods:**

Data from 1,411 people aged 60+ in Shaanxi, China was used in this study. The OLS estimate was used to analyze the impact of using CHSA on well-being of older adults. Then, the instrumental variable estimate was further hired to examine associations among variables to address the potential endogeneity concerns. The heterogeneity among disabled and non-disabled group was also estimated.

**Results:**

For objective well-being, the mental health (*β* = 0.44) and financial well-being (*β* = 0.59) of older people using CHSA were significantly higher than those not using any service. The impact on objective well-being were more significant in non-disabled group. For subjective well-being, life meaning (*β* = 0.54) and security (*β* = 0.72) were positively associated with CHSA utilization, while independence index (*β* = −0.33) was negatively related with CHSA utilization. The subjective well-being of disabled group using services increased more than non-disabled group.

**Conclusion:**

Findings underscore the positive, albeit group-selective, role of CHSA in improving well-being of older people in China and imply the necessity of high-quality development of CHSA and the targeted differentiation strategy.

## Introduction

In 2016, ‘Outline of the Healthy China 2030 Plan’ had highlighted the healthcare concerns of focus groups like older people, which matters a lot to the well-being of an individual and the society (1). As the country with the largest aged population in the world, China is facing great care services burden for the aged, especially health services. The poor accessibility to institutional medical resources is a national concern in China, no exception for older people (2). Whilst older adults are more likely to obtain quality health services in nursing homes, only a minority of them afford the nursing home resources to solve their care demands, even it does not match their living preference (3, 4). As most Asian countries, families are the basic part of Chinese care services system for the aged, but most of them are incapable of meeting the multi-leveled health demands, which is a potential barrier for older people, especially those having special difficulties like chronic diseases, disabilities or frailty, to aging in place (5, 6).

Under this circumstance, the superiority of the community in supplying health services for the aged has aroused widely concern. Since the 1990s, the Chinese government has promoted the development of the community as an important carrier for connecting and integrating multiple public resources, and seized the momentum to develop community care service for the aged (CCSA) (7, 8). CCSA provides in local communities are aimed to satisfy various care service needs in terms of daily care support, healthcare, recreational and spiritual needs of the community-dwelling older people and share the pressure of family care. Thereinto, health services have been embedded in the development of CCSA. It is believed that the development of community health services for the aged (CHSA) has strong rationality, as it supports older adults to age in place with more accessible and professional health services (9). CHSA have diverse service modes and providers. For instance, the primary health care agencies like community health care centers or stations in urban area or village clinics in rural area are one of the main medical service providers of CHSA. With the support of the government, the CCSA centers could connect and integrate social healthcare resources and provide services like free medical examination and health documentation, as well as door-to-door medical services, escorting to hospital and emergency assistance with low price. Also, the senior or health associations, voluntary organizations and universities are included with services like free health lecture and local pharmacies are encouraged to offer free medication guidance. Based on the rationality of this system, the Chinese government has put great emphasis on development of CHSA to improve the accessibility of health resources and respond to the rapid growth of healthcare demands of older people (10, 11). While CHSA have obtained great system rationality and top-down policy support, the service utilization rate is surprisingly low (12). One of the key reasons is that benefits of using these services are still unclear and older people have not built confidence in them yet. That is, the bottom-up identity from older people for the development of CHSA is deficient. Evaluations on benefits of public interventions on individuals is the important basis for shaping public identity and decisions making on the allocation of limited public resources (13), thus calling for further empirical evidence on the outcome benefits of CHSA.

Previous studies on this issue were mainly focus on CCSA, and the health-related outcomes dominate the research on benefits of CCSA. It is reported that for older adults in western countries or in China, the provision or utilization of CCSA is effective in reducing hospitalization risks and in delaying institutionalization (14), decreasing health vulnerability and contributing to health (15, 16). For the frail and disabled older adults, using CCSA also has protective effect on their physical and mental health (17, 18). Few studies had focused on outcome benefits of CHSA on older people, even it is a more rigid and irreplaceable service compared with other CCSA categories like daily care services (19).

With increasing awareness that for evaluations conducted in the aged care sector, effectiveness is best determined through the measurement of outcomes with broad scopes (20), health that reduces outcome benefits into a single dimension seem to be no longer appropriate as they may underestimate the effect of healthcare resources on overall well-being of older people (21, 22). Thus, well-being, referring to the set of individuals’ welfares (23), is highly recommended and widely accepted for evaluations on the outcomes of health and social care interventions for the aged. Well-being can be divided into objective well-being and subjective well-being, by distinguishing between functional dimensions and subjective perception dimensions (23). In general, objective well-being refers to functions or capabilities independent of individual preferences (i.e., health condition and finance capability), while subjective well-being refers to subjective feeling or evaluation on life based on individual standards. Previous studies on well-being measurement mainly focuses on subjective well-being (24, 25).

Till now, no empirical evidence on the impact of using CHSA on well-being of older adults from both objective and subjective dimensions has been provided. Additionally, CHSA is developed for all community-dwelling older people and is required to incline to older people with special difficulties. In particular, disability is a prominent problem in older people, which decreases a person’s ability to carry out activities of daily living, and further places a strain on family members and calls for healthcare support urgently (26). So far, effect of CHSA utilization on the disabled older adults has not been fully studied in China. Therefore, the aim of this study is to examine the impact of CHSA utilization on objective and subjective well-being of older adults and further test the heterogeneity between disabled and non-disabled group. To address this potential endogeneity issue, the instrumental variable model is applied in this study. The current study will provide further empirical evidence on the bottom-up identity for CHSA development, forming pulling force for older people to use these services and supporting policy makers to implement more optimal allocate strategy with limited healthcare resources.

## Methods

### Participants and sampling

The data used in this study were obtained through the survey organized by Xi’an Jiaotong University from October 2019 to January 2020. This survey was conducted in China’s Shaanxi Province, which is ideal for our study, as it is characterized by rapid population aging and ranked as moderate in economic development. Also, Shaanxi is one of the CCSA reform pilot provinces in China and was awarded the outstanding case. A stratified sampling method was employed to yield the sample in this study. We conducted a pre-research in three representative cities first, namely, Baoji, Yan’an, and Hanzhong. Then, the survey was fully conducted in all 10 cities in Shaanxi. In each city, the isometric random sampling method was used to select two or three counties (districts). Three communities equipped with integrated service centers of CCSA were selected according to their administrative divisions. Finally, we randomly selected about 20 participants in each community if they (1) were aged ≥60 years, (2) were able to communicate easily or communicate with investigators’ assistance, and (3) volunteered to participate in the study.

### Data collection

Data were collected through face-to-face interviews conducted by well-trained investigators from the research team. In this process, our investigators asked the participants and completed questionnaires based on their answers. Three categories of indicators were included in this survey: (a) utilization of CCSA (including CHSA); (b) objective and subjective well-being of older adults; and (c) socio-demographic questions. A total of 1,479 older people took part in this survey and 1,411 older people were included in final sample after filtering questionnaires with amounts of indicators data missing. Informed consent was obtained from all participants prior to the survey. Study protocols and consent forms were approved by the medical ethics committee of Health Science Center of Xi’an Jiaotong University (approval number 2016-416). All participants provided informed consent to participate in the study.

### Measures

#### Outcome variable

The dependent (outcome) variable, well-being of older adults, was divided into objective well-being and subjective well-being in this study. Objective well-being was assessed by constructing physical health well-being index (PHW index), mental health well-being index (MHW index), social health well-being index (SHW index) and financial well-being index (FW index). The detailed measurement of objective well-being indexes was shown in Appendix Table 1.

Based on dimensions and indicators of previous subjective well-being scales for older people (13, 21, 23), eight subjective well-being indicators, including life satisfaction, feeling hopeful, personal safety, independence, achievement in life, feeling useful, feeling respected and future safety, were selected from our questionnaire. Life meaning index, security index and independence index were extracted by principal component analysis (PCA) from eight indicators (see Appendix Table 2) and the three indexes were measured by calculating the average value of common indicator(s). All the three subjective well-being indexes were standardized in regression. To all indexes, the higher the score, the higher the degree of well-being.

#### Independent variable

CHSA utilization was the dependent variable in this study. Five CHSA items, including health management (i.e., health lecture and medical examination), door-to-door medical services (i.e., therapy and traditional Chinese medicine health care), medical services in community health center, escorting to hospital and emergency assistance, were listed in the questionnaire and participants were asked if they had used each of them. Once a respondent said that he/she had used one or more service items, CHSA utilization was defined as Yes and a value of 1 was assigned. If a respondent did not use any service, it was defined as No and a value of 0 was assigned.

#### Control variables

Age, gender, educational level, marital status, living with children, Hukou location, family care and weekly exercise were included as control variables based on prior researches and expectation that they would affect well-being of older adults. These control variables also strengthen the instruments’ exogeneity as they reduce the effect of the error term on the instrument.

#### Instrumental variable

While using CHSA may have a significant impact on well-being of older adults, the decision on services use of older people is also influenced by their well-being status. For instance, older people with better physical health status may be less likely to use health services (27, 28). To address the potential endogenous issue and accurately estimate the impact of CHSA utilization on well-being, the prevalence of using services in a community (PUSC) was chosen as the instrument in this study to solve the potential endogeneity bias. PUSC is measured by calculating the proportion of older people (not including the participant) in the community who have used services. It was expected to positively drive or inhibit the CHSA utilization of the participant (relevance), but not to affect well-being of the participant and error term directly (exogeneity).

### Statistical analysis

Simple descriptive statistics were estimated to illustrate the characteristics of our participants. To estimate the impact of CHSA utilization on well-being of older adults, the ordinary least square (OLS) estimate was first conducted. Given the potential bias caused by endogeneity, instrumental variable (IV) estimate was further used. The two-stage least square (2SLS) approach is one of the most common IV approaches. In this study, our initial stage is to test the exclusion restrictions of instrument and it is presented by the equation as follows:


(1) $$E=\alpha_{0}+\alpha_{1}IV+\alpha X+\varepsilon$$

Where E is our endogenous regressor (CHSA utilization), $\alpha_{i}$ is the estimated parameter coefficients, IV is the instrument (PUSC), X is a vector of all control variables and ε is the error term. CD Wald F statistic was conducted in the first stage to avoid weak instrument (29).

In the second stage, the regression between predicted CHSA utilization obtained from Equation 1 and well-being of older adults is estimated by the Equation 2 as follows:


(2) $$Y=\beta_{0}+\beta_{1}\hat{E}+\beta X+\vartheta$$

Where Y is the unbiased estimation of our dependent variable (well-being of older adults), $\beta_{j}$ is the estimated parameter coefficients, $\hat{E}$ is the “purged” endogenous variable, X is a vector of all other control variables and ϑ is the error term.

To further analyze the well-being outcomes differences of using CHSA between disabled and non-disabled older people, heterogeneity test on the impact of CHSA utilization on well-being of disabled and non-disabled groups was further conducted in this study. All statistical analyses were conducted in Stata version 15 (StataCorp, College Station, TX, USA).

## Results

### Characteristics of participants

A total of 1,411 older people fully completed the survey. The baseline characteristics of participants are reported in Table 1. The age of participants ranged from 60 to 97, with 71.54 on average. 49.04% of older people were female and 44.15% of them had the educational level of primary school or lower. Most participants (70.80%) were non-single. 30.47% of them lived with children and 55.35% were urban residents. Most participants had pension insurance (95.39%) and health insurance (97.87%). Nine hundred and fifty two participants reported receiving family care and 35.65% had regular exercise weekly. 16.16%of participants reported that they had used CHSA.

**Table 1:** Descriptive statistics of basic characteristics of participants.

Variables	Definition	*N* (%)/Mean (SD)
Age (years)	–	71.54 (8.07)
	Range	60, 97
Gender	Female = 1	692 (49.04%)
	Male = 0	719 (50.96%)
Educational level	Primary school or lower = 1	623 (44.15%)
	Secondary school = 2	396 (28.07%)
	Secondary school or higher = 3	392 (27.78%)
Marital status	Single = 1	412 (29.20%)
	Non-single = 0	999 (70.80%)
Living with children	Yes = 1	430 (30.47%)
	No = 0	981 (69.53%)
Hukou location	Urban = 1	781 (55.35%)
	Rural = 0	630 (44.65%)
Family care	Yes = 1	1,346 (95.39%)
	No = 0	65 (4.61%)
Weekly exercise	Yes = 1	1,381 (97.87%)
	No = 0	30 (2.13%)
CHSA utilization	Yes = 1	952 (67.47%)
	No = 0	459 (32.53%)

### IV estimate results—first stage results

To overcome potential endogeneity problems, 2SLS model was estimated in this study. First of all, the first stage of the 2SLS process was estimated to confirm the relevance of the instruments on CHSA utilization. According to the results of first stage of 2SLS model (Table 2), PUSC is a positive explanatory factor to CHSA utilization, showing a significant relationship between the instrument and the endogenous regressor. The results indicated the rationality of the instrument choice. The CD Wald F was 231.881 in this study, easily exceeding the threshold value 10 and we can conclude that PUSC is not an invalid or weak instrument.

**Table 2:** Results of first stage of 2SLS models in full samples.

Variables	CHSA utilization
PUSC	0.89**
	(0.06)
Age	0.01
	(0.02)
Gender (Ref. Male)	0.01
	(0.02)
Educational level (Ref. Primary school or lower)	
Secondary school	−0.01
	(0.01)
Secondary school or higher	0.05
	(0.01)
Marital status (Ref. Non-single)	0.02
	(0.02)
Living with family (Ref. No)	0.01
	(0.02)
Hukou location (Ref. Rural)	−0.02
	(0.01)
Family care (Ref. No)	−0.01
	(0.00)
Weekly exercise (Ref. No)	0.03*
	(0.00)
Constant	0.03
	(0.06)
*N*	1,411
R-squared	0.205
CD Wald F	231.881

Robust standard error in parentheses; ***p < 0.01, **p < 0.05, *p < 0.1.

### Effect of CHSA utilization on objective well-being

Table 3 shows the results of 2SLS estimate of CHSA utilization on objective well-being. The OLS estimate is also reported for comparison. The OLS results indicated a significant positive relationship between using CHSA and PHW index and FW index. Everything else being equal, a 1% increase in CHSA utilization is associated with a 12% increase in the PHW index, and a 17% increase in the FW index. After addressing the endogeneity, the 2SLS estimate showed different results, indicating that using CHSA has a significantly positive impact on MHW index and FW index. The results of 2SLS models showed that, everything else being equal, a 1% increase in CHSA utilization was correlated with a 44 and 59% increase in MHW index and FW index of older people.

**Table 3:** The OLS and 2SLS estimate of CHSA utilization on objective well-being in full samples.

Variables	OLS			2SLS				
	PHW index	MHW index	SHW index	FW index	PHW index	MHW index	SHW index	FW index
CHSA utilization	0.12***	0.10	0.08	0.17***	0.01	0.44***	−0.00	0.59***
	(0.04)	(0.07)	(0.05)	(0.05)	(0.04)	(0.13)	(0.05)	(0.12)
Age	−0.05**	0.08**	−0.10***	0.04	−0.05***	0.07***	−0.10***	0.04**
	(0.02)	(0.04)	(0.03)	(0.03)	(0.01)	(0.01)	(0.01)	(0.02)
Gender (Ref. Male)	−0.09**	0.09*	0.01	0.14***	−0.08***	0.08	0.01	0.13***
	(0.03)	(0.05)	(0.04)	(0.04)	(0.02)	(0.10)	(0.02)	(0.02)
Educational level (Ref. Primary school or lower)								
Secondary school	0.03	0.00	0.08*	0.11**	0.03	0.01	0.08***	0.12***
	(0.04)	(0.07)	(0.05)	(0.05)	(0.02)	(0.02)	(0.02)	(0.03)
Secondary school or higher	0.19***	0.25***	0.34***	0.24***	0.19***	0.24***	0.35***	0.23***
	(0.04)	(0.07)	(0.05)	(0.05)	(0.01)	(0.00)	(0.02)	(0.06)
Marital status (Ref. Non-single)	−0.15***	−0.23***	−0.02	0.01	−0.15***	−0.24***	−0.02	−0.00
	(0.04)	(0.07)	(0.05)	(0.05)	(0.00)	(0.06)	(0.02)	(0.07)
Living with family (Ref. No)	−0.03	0.15***	0.05	−0.02	−0.03	0.16***	0.05**	−0.02**
	(0.04)	(0.06)	(0.04)	(0.04)	(0.02)	(0.02)	(0.02)	(0.01)
Hukou location (Ref. Rural)	0.01	0.25***	−0.10**	0.12***	0.01	0.25***	−0.10***	0.12***
	(0.04)	(0.06)	(0.04)	(0.04)	(0.01)	(0.03)	(0.03)	(0.02)
Family care (Ref. No)	−0.04	0.05	−0.01	−0.11**	−0.04**	0.07	−0.02	−0.08***
	(0.04)	(0.06)	(0.05)	(0.05)	(0.02)	(0.05)	(0.02)	(0.03)
Weekly exercise (Ref. No)	0.07**	0.19***	0.20***	0.08*	0.08***	0.18***	0.20***	0.06***
	(0.03)	(0.05)	(0.04)	(0.04)	(0.00)	(0.01)	(0.01)	(0.01)
Constant	0.01	0.04	−0.03	−0.46***	0.04	−0.03	−0.02	−0.55***
	(0.14)	(0.22)	(0.17)	(0.16)	(0.08)	(0.07)	(0.10)	(0.04)
*N*	1,411	1,411	1,411	1,411	1,411	1,411	1,411	1,411
R-squared	0.046	0.078	0.070	0.072	0.042	0.062	0.069	0.031

Robust standard error in parentheses; ***p < 0.01, **p < 0.05, *p < 0.1.

### Effect of CHSA utilization on subjective well-being

Table 4 presents the results of IV estimate of CHSA utilization on subjective well-being. The OLS results indicated significantly positive impact of using CHSA on life meaning index (*β* = 0.13) and security index (*β* = 0.14), while the significant relationship between using CHSA and independence index was not observed. With the endogeneity problem considered, the 2SLS results also indicated a positive impact of using CHSA on life meaning index and security index. Everything else being equal, a 1% increase in CHSA utilization is associated with a 54% increase in the life meaning index, and a 72% increase in the security index, showing a larger impact than the estimation results of OLS models. Unlike the results of OLS models, 2SLS models also showed a negative impact on independence index (*β* = −0.33). It is likely that the OLS models may underestimate the impact of CHSA utilization on subjective well-being of older adults and the endogeneity bias should not be ignored.

**Table 4:** The OLS and 2SLS estimate of CHSA utilization on subjective well-being in full samples.

Variables	OLS			2SLS		
	Life meaning	Security	Independence	Life meaning	Security	Independence
CHSA utilization	0.13*	0.14*	0.00	0.54**	0.72***	−0.33***
	(0.07)	(0.07)	(0.07)	(0.23)	(0.17)	(0.13)
Age	0.01	0.12***	−0.03	0.01	0.12***	−0.03***
	(0.04)	(0.04)	(0.04)	(0.02)	(0.01)	(0.00)
Gender (Ref. Male)	0.06	0.14**	−0.09*	0.04***	0.11***	−0.08***
	(0.05)	(0.05)	(0.05)	(0.01)	(0.04)	(0.02)
Educational level (Ref. Primary school or lower)						
Secondary school	0.03	0.00	0.14**	0.04***	0.02***	0.13***
	(0.07)	(0.07)	(0.07)	(0.00)	(0.00)	(0.05)
Secondary school or higher	0.25***	0.07	0.15**	0.24***	0.06***	0.15***
	(0.07)	(0.07)	(0.07)	(0.03)	(0.02)	(0.03)
Marital status (Ref. Non-single)	0.06	−0.02	0.08	0.05	−0.03	0.09**
	(0.07)	(0.07)	(0.07)	(0.04)	(0.06)	(0.04)
Living with family (Ref. No)	−0.09	0.04	−0.39***	−0.09***	0.05*	−0.39***
	(0.06)	(0.06)	(0.06)	(0.00)	(0.03)	(0.07)
Hukou location (Ref. Rural)	0.03	0.11*	−0.14**	0.03**	0.11***	−0.14***
	(0.06)	(0.06)	(0.06)	(0.01)	(0.02)	(0.01)
Family care (Ref. No)	0.11*	−0.03	0.11*	0.14***	0.01	0.08
	(0.06)	(0.06)	(0.06)	(0.01)	(0.02)	(0.06)
Weekly exercise (Ref. No)	0.20***	0.11*	0.09	0.19***	0.09***	0.10***
	(0.06)	(0.06)	(0.06)	(0.02)	(0.01)	(0.01)
Constant	−0.58***	−0.25	0.09	−0.67***	−0.37***	0.16***
	(0.22)	(0.22)	(0.22)	(0.06)	(0.10)	(0.01)
*N*	1,411	1,411	1,411	1,411	1,411	1,411
R-squared	0.038	0.029	0.040	0.015	0.022	0.025

Robust standard error in parentheses; ***p < 0.01, **p < 0.05, *p < 0.1.

### Heterogeneity test

To better understand the well-being outcomes differences of using CHSA between disabled and non-disabled older people, heterogeneity test on the impact of CHSA utilization on well-being of disabled and non-disabled groups was further analyzed by using 2SLS estimate. In this study, disabled older people are defined as those reporting as needing any assistance(s) on one or more items of activities of daily living (ADL) (including bathing, dressing, using the toilet, indoor transferring, controlling urination and defecation, and eating) (30).

The results of the first stage of the 2SLS estimate are presented in Appendix Table 3. It shows that there is a significant relationship between the instrument and the endogenous regressor among both disabled and non-disabled group, with the instrument (PUSC) are significant at the 10% level. Besides, the CD Wald F was 31.864 among the disabled group and 96.810 among the non-disabled group, exceeding the threshold value 10 and passing the weak instrument test.

According to results of the second stage of the 2SLS estimate, for the 227 disabled older people, CHSA utilization had significantly positive impact on MHW index (*β* = 0.36), SHW index (*β* = 0.31), FW index (*β* = 0.69), life meaning index (*β* = 1.48) and security index (*β* = 1.03) of older people, while the impact of CHSA utilization on independence index (*β* = −1.50) was significantly negative and no significant relationship between CHSA utilization and PHW index was observed.

For the 1,184 non-disabled older people, CHSA utilization had significantly positive impact on all well-being indexes, except for SHW index (*β* = 0.09) and independence index (*β* = −0.12). Comparatively, the impact of CHSA utilization on objective well-being, particularly the physical and mental health well-being in non-disabled group were stronger than in disabled group. Conversely, the result indicated a 0.72 and 0.89 increase in life meaning index and security index of non-disabled older people using CHSA, significantly weaker than disabled older people (Table 5).

**Table 5:** Heterogeneous effect of CHSA utilization on well-being of disabled and non-disabled group.

Variables		PHW index	MHW index	SHW index	FW index	Life meaning	Security	Independence
Disabled group	CHSA utilization	0.18	0.36***	−0.31	0.69***	1.48***	1.03***	−1.50***
		(0.20)	(0.12)	(0.23)	(0.02)	(0.24)	(0.31)	(0.16)
	CVs	Yes	Yes	Yes	Yes	Yes	Yes	Yes
	Constant	−0.35***	0.43*	−0.34***	−0.41***	−1.60***	−0.74***	−1.79***
		(0.06)	(0.25)	(0.13)	(0.03)	(0.16)	(0.12)	(0.19)
	*N*	227	227	227	227	227	227	227
	R-squared	0.103	0.123	0.082	0.082	0.061	0.060	0.054
Non-disabled group	CHSA utilization	0.24***	0.71***	0.09	0.70***	0.72*	0.89***	−0.12
		(0.07)	(0.23)	(0.11)	(0.03)	(0.41)	(0.27)	(0.25)
	CVs	Yes	Yes	Yes	Yes	Yes	Yes	Yes
	Constant	0.08***	−0.03	−0.06	−0.63***	−0.86***	−0.55***	−0.08
		(0.00)	(0.08)	(0.14)	(0.01)	(0.07)	(0.02)	(0.06)
	*N*	1,184	1,184	1,184	1,184	1,184	1,184	1,184
	R-squared	0.071	0.085	0.087	0.081	0.030	0.037	0.059

CVs, control variables. Robust standard error in parentheses. ***p < 0.01, **p < 0.05, *p < 0.1. The full table of results is provided in Appendix Tables 4, 5.

## Discussion

To our knowledge, this is the first study to examine the impact of CHSA utilization on well-being of older adults from both objective and subjective dimensions. Also, IV estimate was first hired in this issue to address endogeneity concerns, which were obviously neglected in previous studies (31). With PUSC as the instrumental variable, the 2SLS estimate offered a more accurate prediction. It was evidenced that except for PHW index and SHW index, CHSA utilization had a significant impact on all objective well-being and subjective well-being of older adults.

In terms of objective well-being, older people using CHSA had significant increase on their MHW index and FW index, but not on PHW index and SHW index. Previous studies indicated that CHSA could help older adults increase health knowledge and health awareness, thus preventing the onset of disability, chronic diseases and depressive symptoms (32, 33). Our findings were only partly consistent with previous studies, with no significant association between CHSA and physical health observed. There is no denying that the implement of community-based health services policy is effective on promoting accessibility of professional health interventions for community-dwelling older people in China (34). However, China is still suffering from insufficient medical resources, especially the primary medical care in the community level. So far, CHSA in China mainly focuses on basic service items like free medical examination and medication guidance, which are hard to improve physical function of older people in a short time. For social health well-being of older adults, numerous studies had proved that social engagement is an important predictor of physical and mental health of older people (35) and conversely, better physical function is related with higher social integration and less social isolation (36). However, as mentioned above, CHSA utilization was not effective on improving physical health and it may consequently lead to the ineffective impact of CHSA utilization on social health well-being. For financial well-being, using CHSA is unlikely to boost income directly though, it works on reducing expenditures on health as a low-cost substitution for basic medical services in large hospital (37).

In terms of subjective well-being, there were significantly positive correlations between CHSA utilization and life meaning and security, while the correlation between CHSA utilization and independence was negative. Life meaning reflects the satisfaction or recognition on life or individual value of older people. As discussed above, CHSA utilization could potentially improve older people’s health and financial well-being, thus increasing their perceived life satisfaction and value (38). For the positive effect of CHSA utilization on security index, CHSA could facilitate older people’s access to healthcare interventions, shortening the time and distance between medical resources and community-dwelling older people greatly and relieving their anxiety and insecurity about health (39). Also, using CHSA works on decreasing older people’s health worries and financial pressure, and further enhances their confidence on later life. Independence index reflects whether older people feel free to do what they want to do or make their decision, which is influenced by their physical function and autonomy (23). For the negative relationship between CHSA utilization and independence index, older adults using CHSA may have some health problems that impede them to live their life independently. Another possible explanation is that even using CHSA works on functional improvement of older people, they may still be restrained by factors like family life or money to live life freely.

As expected, the impact of CHSA utilization on well-being of older adults was different between disabled and non-disabled older people. Comparatively, impact of CHSA utilization on objective well-being in non-disabled group seemed to be stronger. Specifically, using CHSA was significantly and positively correlated with physical and mental health well-being among the non-disabled older adults, while no significant correlation between CHSA utilization and physical health well-being was observed among disabled older people. A possible explanation is that, for older people without serious health problems, CHSA may be benefit on their health promotion and improve their physical condition. While owning to the unbalanced and inadequate medical resources allocation in China, the existing CHSA are far from meeting the high-level professional healthcare demands of disabled older people and making essential improvement on their physical health (33). Furthermore, the positive impact on subjective well-being were relatively stronger in disabled group. With impaired self-care ability, disabled older people are suffering from multiple life difficulties and their baseline level of subjective well-being is inevitably lower than non-disabled people, thus they may be more sensitive to the benefits from using CHSA and obtain higher life satisfaction or life security. In particular, given the limited services quality, it is unlikely for older adults with ADL limitations to recover to a good functional condition for living independently, thus the negative relationship between CHSA utilization and independence index of disabled older people is not surprising.

## Policy implications

This study extends upon earlier findings and has important implications for policy and practice. For a long time, CHSA has been viewed as an important coping strategy for the increasing healthcare demands of older people and obtained great top-down policy support in China based on its system rationality. However, the evaluation of outcome benefits of these services has been overlooked. The current study provides empirical evidences on the positive impact of CHSA utilization on well-being of older adults and these findings are unbiased, implying that the role of CHSA as a policy tool in China is effective and providing the important bottom-up identity basis for further development. With these findings, policy advocacy about the benefits of using CHSA should be more widely promoted among older adults to encourage engagement and utilization. Meanwhile, more efforts should be taken to increase the accessibility and quality of health services for community-dwelling older people. For example, the government could take the lead to input more public investments and attract more social funds to support the establishment and upgrade of community healthcare facilities. More focus should be shifted to the professional training of healthcare personnel and offering high-quality professional treatments on diseases of old age (40). Also, the quality evaluation on CHSA from public perspective could be taken as an important indicator in community performance assessment. Moreover, the results revealed the difference between disabled and non-disabled group. It indicated the unmet needs for health services of disabled older people and implied the differentiation strategy on CHSA in China. A more targeted and high-quality provision system of CHSA that meet more accurate demands and incentivizes deeper engagement may be valuable.

## Limitations

Our study has some limitations that must be considered in the interpretation of the findings. First, this study was a cross-sectional study with a focus on one province. While Shaanxi Province is an ideal representative area for this study, it might not be able to reflect the longitudinal perspectives and capture all regional characteristics of the impact of CHSA utilization on well-being of older adults in China. Secondly, CHSA have diverse contents while they were viewed as a whole in this study, which may obscure the specific benefits of different CHSA contents. Thirdly, this study focused on the heterogeneity between disabled and non-disabled group, while the sample size of disabled older people was only 227, which may lead to some statistical bias and weak stability of the results. Also, more specific groups like different age groups, frailty groups or urban–rural groups should be further studied to support the diverse development and targeted provision of CHSA.

## Conclusion

Our findings indicated that CHSA utilization had a positive impact on objective well-being like mental health and financial well-being, and on subjective well-being like life meaning and security of community-dwelling older people, while the relationship between CHSA utilization and independence was negative. Comparatively, the positive impact on objective well-being was stronger in non-disabled group, while the positive impact on subjective well-being relatively higher in non-disabled group. These findings recognized the effective role of CHSA as a policy tool in China by evaluating its benefits on promoting well-being of older adults, forming the bottom-up identity basis for further development.

## Data Availability

The raw data supporting the conclusions of this article will be made available by the authors, without undue reservation.
