# Supplementary material for: From rationality to identity: the impact of using community health services for the aged on the well-being of older adults in China
Source: Front Public Health. 2024 Sep 24;12:1447217. doi: 10.3389/fpubh.2024.1447217 (PMC11458453; doi:10.3389/fpubh.2024.1447217)
Supplement: Supplementary file 1 [file Table_1.DOCX]

Supplementary Material

Appendix Table 1. Measurements of objective well-being

| Variables | Measurement |
| --- | --- |
| PHW Index | Standardizing continuous indicator of “physical health status” and binary indicator of “having hospitalization history in the past year”, setting weight for two indicators averagely and summing them up. |
| MHW Index | 1-standardized score of the CESD-10. |
| SHW Index | Standardizing continuous indicators of “social isolation” and “the number of social activities in last month”, setting weight for two indicators averagely and summing them up. |
| FW Index | Standardizing continuous indicators of “living standard”, “financial stress” and “the proportion of non-medical expenditure in total expenditure in the past year”, setting weight for three indicators averagely and summing them up. |

Note: Physical health status was scored from 1 (very bad) to 5 (very good) by our investigators according to participants’ thinking ability and mobility. Social isolation and living standard were scored from 1 (very bad) to 5 (very good) by our investigators according to participants and their family members’ description.

Appendix Table 2. Exploratory factor analysis of eight subjective well-being indicators

| Indicators | Factors | | |
| --- | --- | --- | --- |
|  | F1(Life meaning) | F2 (Security) | F3 (Independence) |
| Life satisfaction | 0.510 |  |  |
| Feeling hopeful | 0.581 |  |  |
| Personal safety |  | 0.555 |  |
| Independence |  |  | 0.592 |
| Achievement in life | 0.432 |  |  |
| Feeling useful | 0.535 |  |  |
| Feeling respected | 0.385 |  |  |
| Future safety |  | 0.596 |  |

Note: Life satisfaction was measured by the question “I feel satisfied with my life as a whole”. Feeling hopeful was measured by the question “I feel full of hope to each day”. Personal safety was measured by the question “I feel safe with my present life”. Independence was measured by the question “I feel free to do the things I want”. Achievement in life was measured by the question “I feel satisfied with my achievement in life”. Feeling useful was measured by the question “I feel useful about myself”. Feeling respected was measured by the question “I feel respected by people around”. Future safety was measured by the question “I have no worries about my life security in the future”. Each indicator is rated on a five-point Likert scale from strongly disagree = 1 to strongly agree = 5.

Appendix Table 3. Results of first stage of 2SLS models in disabled group and non-disabled group

| Variables | Disabled group | Non-disabled group |
| --- | --- | --- |
|  | CHSA utilization | CHSA utilization |
| PUSC | 0.80* | 0.88* |
|  | (0.13) | (0.10) |
| Age | -0.08 | 0.02 |
|  | (0.04) | (0.01) |
| Gender (Ref. Male) | 0.04 | -0.01 |
|  | (0.01) | (0.02) |
| Educational level (Ref. Primary school or lower) |  |  |
| Secondary school | 0.06 | -0.01** |
|  | (0.07) | (0.00) |
| Secondary school or higher | -0.07 | 0.08** |
|  | (0.10) | (0.01) |
| Marital status (Ref. Non-single) | 0.05 | 0.00 |
|  | (0.06) | (0.00) |
| Living with family (Ref. No) | -0.06** | 0.00 |
|  | (0.00) | (0.02) |
| Hukou location (Ref. Rural) | 0.07 | -0.02 |
|  | (0.11) | (0.05) |
| Family care (Ref. No) | -0.12 | 0.01 |
|  | (0.04) | (0.01) |
| Weekly exercise (Ref. No) | -0.02 | 0.04** |
|  | (0.03) | (0.00) |
| Constant | 0.62** | 0.01 |
|  | (0.05) | (0.06) |
| N | 227 | 1184 |
| R-squared | 0.242 | 0.196 |
| CD Wald F | 96.810 | 31.864 |

Appendix Table 4. The 2SLS estimate of CHSA utilization on well-being in disabled group

| Variables | PHW index | MHW index | SHW index | FW index | Life meaning | Security | Independence |
| --- | --- | --- | --- | --- | --- | --- | --- |
| CHSA utilization | 0.18 | 0.36*** | -0.31 | 0.69*** | 1.48*** | 1.03*** | -1.50*** |
|  | (0.20) | (0.12) | (0.23) | (0.02) | (0.24) | (0.31) | (0.16) |
| Age | 0.03 | 0.11** | -0.00 | 0.08*** | 0.22*** | 0.19*** | 0.24*** |
|  | (0.02) | (0.05) | (0.05) | (0.01) | (0.04) | (0.05) | (0.09) |
| Gender (Ref. Male) | -0.08*** | 0.15 | -0.04** | -0.14*** | 0.00 | -0.16*** | 0.06 |
|  | (0.01) | (0.13) | (0.02) | (0.04) | (0.02) | (0.05) | (0.04) |
| Educational level (Ref. Primary school or lower) |  |  |  |  |  |  |  |
| Secondary school | 0.03 | -0.27** | 0.05 | -0.13*** | -0.03 | -0.19*** | -0.31 |
|  | (0.05) | (0.11) | (0.13) | (0.02) | (0.10) | (0.02) | (0.37) |
| Secondary school or higher | 0.22*** | 0.16 | 0.31*** | 0.22*** | 0.31 | 0.36** | -0.32 |
|  | (0.01) | (0.24) | (0.10) | (0.07) | (0.26) | (0.14) | (0.40) |
| Marital status (Ref. Non-single) | -0.12*** | -0.57*** | -0.11*** | -0.18*** | -0.29*** | -0.36* | -0.09 |
|  | (0.04) | (0.07) | (0.03) | (0.02) | (0.02) | (0.19) | (0.07) |
| Living with family (Ref. No) | 0.03 | 0.03 | 0.03 | -0.07 | 0.10 | 0.01 | -0.26* |
|  | (0.05) | (0.06) | (0.07) | (0.06) | (0.11) | (0.08) | (0.16) |
| Hukou location (Ref. Rural) | -0.07 | 0.29*** | 0.01 | 0.15 | 0.13 | 0.13 | 0.37*** |
|  | (0.06) | (0.05) | (0.09) | (0.10) | (0.14) | (0.10) | (0.09) |
| Family care (Ref. No) | -0.13*** | -0.12*** | -0.10*** | -0.11*** | 0.19 | -0.13*** | 0.23*** |
|  | (0.04) | (0.01) | (0.02) | (0.01) | (0.13) | (0.03) | (0.02) |
| Weekly exercise (Ref. No) | 0.20*** | 0.36*** | 0.25*** | 0.39*** | 0.36*** | 0.24*** | 0.19*** |
|  | (0.00) | (0.01) | (0.01) | (0.01) | (0.05) | (0.04) | (0.05) |
| Constant | -0.35*** | 0.43* | -0.34*** | -0.41*** | -1.60*** | -0.74*** | -1.79*** |
|  | (0.06) | (0.25) | (0.13) | (0.03) | (0.16) | (0.12) | (0.19) |
| N | 227 | 227 | 227 | 227 | 227 | 227 | 227 |
| R-squared | 0.103 | 0.123 | 0.082 | 0.082 | 0.061 | 0.060 | 0.054 |

Note: Robust standard error in parentheses; *** p<0.01, ** p<0.05, * p<0.1.

Appendix Table 5. The 2SLS estimate of CHSA utilization on well-being in non-disabled group

| Variables | PHW Index | MHW Index | SHW Index | FW Index | Life meaning | Security | Independence |
| --- | --- | --- | --- | --- | --- | --- | --- |
| CHSA utilization | 0.24*** | 0.71*** | 0.09 | 0.70*** | 0.72* | 0.89*** | -0.12 |
|  | (0.07) | (0.23) | (0.11) | (0.03) | (0.41) | (0.27) | (0.25) |
| Age | -0.06*** | 0.08*** | -0.11*** | 0.04*** | -0.00 | 0.13*** | -0.02 |
|  | (0.01) | (0.02) | (0.03) | (0.01) | (0.03) | (0.04) | (0.02) |
| Gender (Ref. Male) | -0.07*** | 0.08 | 0.03* | 0.21*** | 0.09*** | 0.20*** | -0.09** |
|  | (0.01) | (0.10) | (0.02) | (0.02) | (0.02) | (0.05) | (0.05) |
| Educational level (Ref. Primary school or lower) |  |  |  |  |  |  |  |
| Secondary school | 0.01 | 0.03 | 0.05 | 0.11*** | -0.04*** | -0.01 | 0.10*** |
|  | (0.04) | (0.05) | (0.04) | (0.03) | (0.01) | (0.01) | (0.02) |
| Secondary school or higher | 0.17*** | 0.22*** | 0.31*** | 0.15* | 0.12*** | -0.10 | 0.13*** |
|  | (0.06) | (0.06) | (0.06) | (0.09) | (0.04) | (0.08) | (0.03) |
| Marital status (Ref. Non-single) | -0.14*** | -0.17*** | 0.00 | 0.05 | 0.16*** | 0.05*** | 0.15*** |
|  | (0.02) | (0.04) | (0.03) | (0.08) | (0.04) | (0.01) | (0.05) |
| Living with family (Ref. No) | 0.00 | 0.22*** | 0.10*** | 0.04** | -0.00 | 0.13*** | -0.26*** |
|  | (0.04) | (0.02) | (0.03) | (0.02) | (0.07) | (0.01) | (0.00) |
| Hukou location (Ref. Rural) | 0.02*** | 0.21*** | -0.17*** | 0.00 | -0.12*** | -0.01 | -0.36*** |
|  | (0.01) | (0.02) | (0.03) | (0.04) | (0.01) | (0.05) | (0.00) |
| Family care (Ref. No) | -0.03*** | 0.11 | 0.00 | -0.10** | 0.13** | 0.02*** | 0.09 |
|  | (0.01) | (0.07) | (0.03) | (0.04) | (0.06) | (0.00) | (0.10) |
| Weekly exercise (Ref. No) | 0.04*** | 0.12*** | 0.18*** | -0.02*** | 0.11*** | 0.03 | 0.02 |
|  | (0.01) | (0.01) | (0.00) | (0.01) | (0.03) | (0.02) | (0.01) |
| Constant | 0.08*** | -0.03 | -0.06 | -0.63*** | -0.86*** | -0.55*** | -0.08 |
|  | (0.00) | (0.08) | (0.14) | (0.01) | (0.07) | (0.02) | (0.06) |
| N | 1184 | 1184 | 1184 | 1184 | 1184 | 1184 | 1184 |
| R-squared | 0.071 | 0.085 | 0.087 | 0.081 | 0.030 | 0.037 | 0.059 |

Note: Robust standard error in parentheses; *** p<0.01, ** p<0.05, * p<0.1.
